# Pilot Randomized Controlled Trial of an Exercise Program Requiring Minimal In-person Visits for Youth With Persistent Sport-Related Concussion

**DOI:** 10.3389/fneur.2019.00623

**Published:** 2019-06-17

**Authors:** Sara P. D. Chrisman, Kathryn B. Whitlock, Jason A. Mendoza, Monique S. Burton, Ellie Somers, Albert Hsu, Lauren Fay, Tonya M. Palermo, Frederick P. Rivara

**Affiliations:** ^1^Center for Child Health, Behavior and Development, Seattle Children's Research Institute, Seattle, WA, United States; ^2^Department of Pediatrics, University of Washington, Seattle, WA, United States; ^3^Harborview Injury Prevention and Research Center, Seattle, WA, United States; ^4^Department of Orthopedics and Sports Medicine, Seattle Children's Hospital, Seattle, WA, United States; ^5^Department of Sports Physical Therapy, Seattle Children's Hospital, Seattle, WA, United States; ^6^Department of Anesthesiology and Pain Medicine, University of Washington, Seattle, WA, United States

**Keywords:** brain concussion, child, fear-avoidance, pain, exercise, traumatic brain injury, treatment, sport

## Abstract

**Objective:** To evaluate feasibility and acceptability of a sub-threshold exercise program with minimal in-person visits to treat youth with persistent sport-related concussion, and explore efficacy for improving concussive symptoms, health-related quality of life, and fear-avoidance.

**Study design:** We conducted a pilot randomized controlled trial comparing a 6 week sub-threshold exercise program requiring only two in-person visits to active control (stretching) for 12–18 year old youth with persistent sport-related concussion. We measured moderate-to-vigorous physical activity pre- and post-intervention using accelerometry, and increased goals weekly via phone contact. We examined feasibility and acceptability using qualitative interviews. We used exponential regression to model differences in trajectory of concussive symptoms by experimental group, and linear regression to model differences in trajectory of health-related quality of life and fear-avoidance of pain by experimental group.

**Results:** Thirty-two subjects randomized, 30 completed the study (*n* = 11 control, *n* = 19 intervention), 57**%** female. Youth and parents reported enjoying participating in the study and appreciated the structure and support, as well as the minimal in-person visits. Exponential regression modeling indicated that concussive symptoms declined more rapidly in intervention youth than control (*p* = 0.02). Health-related quality of life and fear-avoidance of pain improved over time, but were not significantly different by group.

**Conclusions:** This study indicates feasibility and potential benefit of a 6 week subthreshold exercise program with minimal in-person visits for youth with persistent concussion. Potential factors that may play a role in improvement such as fear-avoidance deserve further study.

## Introduction

Approximately 1.1–1.9 million youth sustain concussions annually (1), and up to 30% have persistent symptoms such as headache, fatigue, and difficulty concentrating for weeks or months (2–5). Persistent concussive symptoms can impact social development, cognitive function, and academic success, and result in greater utilization of sub-specialty care (6). A recent consensus statement called for research into treatments for persistent concussive symptoms (7), as the extant literature provides little guidance. One promising approach is the use of rehabilitative exercise. Individuals who sustain concussions have increased symptoms with exercise, and exercising at a “sub-symptom threshold” level appears to help them return to function more quickly (8, 9). A few studies have reported benefit for such an approach, but have required weekly in-person visits (8–16). Currently no exercise treatments have been designed for concussion that could be completed with minimal in-person visits and therefore be more easily disseminated. We proposed to address this gap, adapting a pre-existing exercise intervention for concussion to be delivered with only two in-person visits, and utilizing weekly check-ins via phone contact with the youth, as has been used effectively in previous studies (17).

Debate also exists regarding how sub-threshold exercise might produce benefit in patients with persistent concussive symptoms. Some have posited (8, 9, 12) that exercise improves cerebrovascular autoregulation, which appears to be impaired following brain injury. Participating in daily exercise at a level below that which exacerbates symptoms could assist with rehabilitation of neurologic adaptations to exercise, thus decreasing exercise-related symptoms occurring secondary to transmission of systemic pressure into the cerebrovascular space.

We conceptualized that exercise interventions for concussion might also have effects on “fear-avoidance” (18–22). The fear-avoidance model is a prominent theoretical model applied to adults and youth to understand processes through which an acute pain experience can become chronic (18–22), According to this model, individuals who perceive pain as threatening and place a catastrophic meaning on the pain experience (e.g., rumination and worry that pain will not go away), develop pain-related anxiety that maintains avoidance, leading to functional disability, depression, and persistence of pain. Pediatric specific models of fear-avoidance include the important role of parent behavioral and psychological responses to the child's pain experience, recognizing that the child's own experiences develop within the familial context (23, 24).

**Figure 1:**
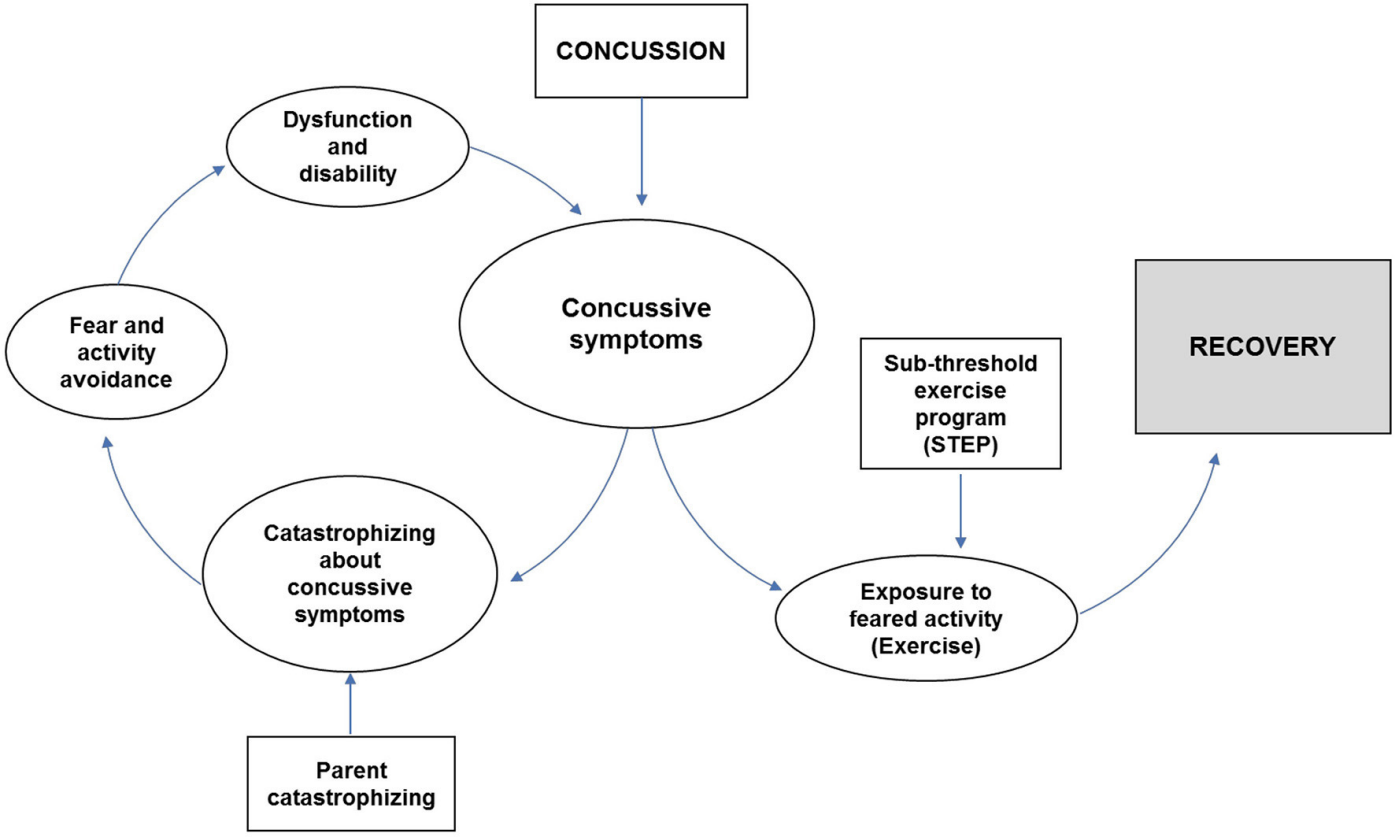
Conceptual model of Fear-avoidance (19), adapted for concussion.

A few authors (25–27) have suggested a role for fear-avoidance in the persistence of concussive symptoms, and we theorized that fear-avoidance might be impacted by sub-threshold exercise programs post-concussion (Figure 1). Patients with acute concussion are initially instructed not to exercise, given concerns about the vulnerability of the injured brain. When symptoms worsen during exercise, concussed patients may fear such increases indicate greater injury. This fear leads them to become anxious about participating in exercise and avoid exercise, bolstering their fear, and generating a cycle of disability. Rehabilitative exercise challenges catastrophic assumptions about exercise, thus decreasing anxiety and avoidance of exercise, and improving function (28). Function in chronic illness states is often conceptualized as health-related quality of life (HRQoL), which can be measured using the Pediatric Quality of Life scale (PedsQL) (29). Prior research has suggested that youth with PPCS have deficits in function that can be measured with the PedsQL (30, 31), and that these deficits can improve with intervention (32).

We conducted a pilot randomized controlled trial (RCT) of a 6-week rehabilitative exercise program requiring minimal in-person visits for youth with persistent concussive symptoms, the Sub-Threshold Exercise Program (STEP). Our goals were to: (a) assess feasibility and acceptability of this approach, (b) collect pilot data regarding the effect of STEP on trajectory of concussive symptoms, and variation by demographic and injury factors (sex, age, prior concussion, time since injury), and (c) explore impact of the intervention on objectively measured moderate-vigorous physical activity (MVPA), standardized measures of fear-avoidance of pain, and health-related quality of life over time.

## Methods

### Recruitment

We recruited youth 12–18 years old from concussion clinics at Seattle Children's Hospital and an on-line portal over a period of ~9 months. The on-line portal consisted of a website with information about the study and targeted advertisements on social media platforms. Inclusion criteria included: (1) diagnosis of sports-related concussion consistent with Zurich definition by a clinician experienced with concussion management (33), (2) at least two concussive symptoms, (3) duration of symptoms 3 weeks−6 months after concussion, (4) no contraindication to performing physical activity, and (5) not currently receiving physical therapy to increase physical activity. Subjects completed a baseline exercise tolerance test (a modified Buffalo Concussion Treadmill Test) (8) and were eligible for the study if symptoms worsened during this test, consistent with prior studies (8, 9, 14). Subjects continued to receive usual care from their referring concussion clinician during the study. We did not collect information about other treatments pursued by subjects during the study. The study was approved by the Seattle Children's Hospital Institutional Review Board and registered at Clinicaltrials.gov, NCT02673112. All youth and parents completed written informed consent.

### Procedures

All subjects completed in-person assessments at study entry (baseline) and 6 weeks later (post-intervention). Subjects were examined by a study physical therapist at baseline to ensure they had no concerning cervical spine or vestibular issues that would preclude exercise. The remainder of the assessments were completed online, including weekly assessments of concussion symptoms during intervention, and surveys at 3 and 6 month follow up. Accelerometer assessments were completed for 5–7 days at baseline and 6 weeks to measure moderate-vigorous physical activity (MVPA) in an objective manner. Youth and parents were provided incentives for completing accelerometry measurements and surveys using gift cards.

### Randomization

Randomization was conducted by the study analyst (K.W.) using random number generation in blocks of four, stratified by age (11–13 and 14–18) and sex. Allocation was weighted 2:1: toward the intervention arm to provide maximum data for feasibility and acceptability. Study arm allocation details were placed into opaque envelopes and chosen sequentially.

### Participants

**Figure 2:**
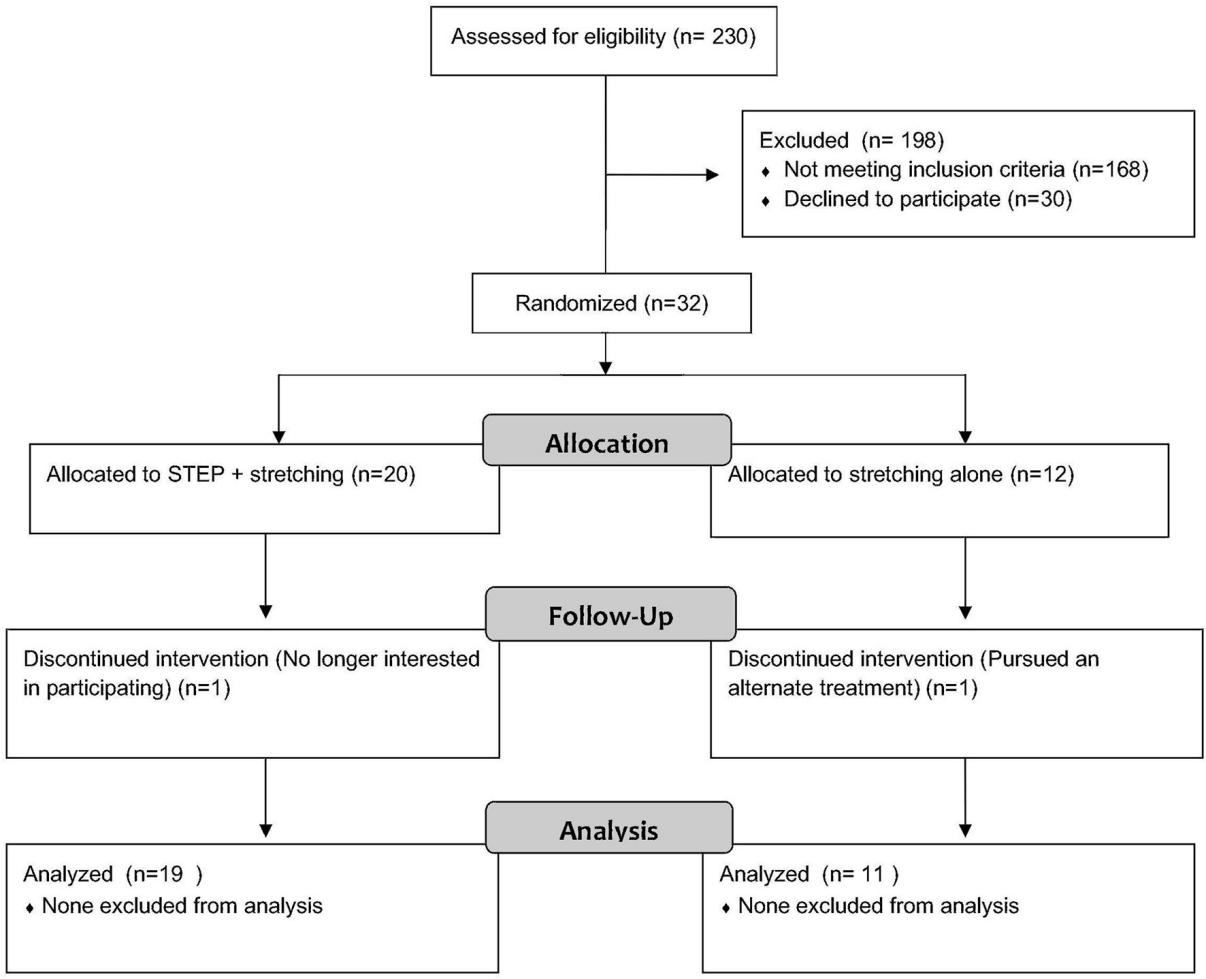
CONSORT flow diagram for Sub-Threshold Exercise Program (STEP), Seattle WA 2016–17.

Two hundred thirty youth were screened, but 69% did not meet inclusion criteria as concussion symptoms had resolved by the time of recruitment contact or they were already receiving physical therapy (Figure 2). Of the 72 patients who met criteria for participation, 45 were initially interested, 10 of whom did not have an increase in symptoms with the treadmill test, and three of whom later declined participation. Thirty-two individuals enrolled and were randomized to intervention or control. Only one of the enrolled participants came from the on-line portal, the remainder were patients at Seattle Children's Hospital. Two subjects discontinued participation in the study, both in the first week of the study, leaving 30 total subjects, 11 control, and 19 intervention.

### Experimental Arms

- **Stretching (Control)**: Individuals randomized to control were given a home stretching program and provided a handout with color illustrations. Stretches required 5–10 min to complete and were completed daily (see Supplementary Material). Research assistants (RAs) called subjects weekly to ensure they were tolerating the exercises.
- **Sub-Threshold Exercise Program (STEP) (Intervention):** Individuals randomized to the intervention were given a 6 week daily home aerobic exercise program. The exercise prescription for participants included specifications for frequency, duration, and intensity as recommended by Howell et al. (34) Frequency was set at daily, with the understanding that youth might miss 1–2 days per week due to scheduling conflicts. Duration of exercise was started at 5–10 min per day greater than MVPA at baseline measured with accelerometry, and increased weekly by 5–10 minutes per/day via phone contact with the RA (for a goal of 60 min/day). Intensity was set at 80% of the heart rate that produced symptoms during the modified Buffalo Concussion Treadmill Test (i.e., “sub-threshold”) (8, 9). Measuring MVPA using actigraphy provided information about quantity of physical activity at baseline, to ensure that the intervention was an increase from a participant's baseline. Utilizing the treadmill test provided information about the intensity of exercise a participant could tolerate, to ensure that the intervention would not provoke symptoms. If symptoms worsened during exercise, HR goal was decreased by 10%. Subjects were provided a wrist-based HR monitor to track heart rate. Participants were instructed to exercise in whatever manner they chose, and most utilized either exercise bike, treadmill, fast walking up an incline/stairs, or calisthenics. Exercise type was allowed to shift during the intervention to adapt to participant preferences and lifestyle.

### Blinding

Physical therapists conducting the modified Buffalo Concussion Treadmill Test were blinded to randomization status. Aside from accelerometry, all other assessments were completed via self-report.

### Demographics, Past Medical History, and Family History

Youth and parents were interviewed together at baseline regarding: (a) date of injury, (b) mechanism, and (c) prior concussion. Parents completed surveys regarding (a) demographics (age, race, socioeconomic status) and (b) past medical history.

### Baseline Exercise Tolerance

The Buffalo Concussion Treadmill Test (35) is a graded treadmill test used to determine heart rate threshold for concussive symptoms. The test was administered in accordance with prior studies (8, 9), beginning with a treadmill speed of 3.3 mph with 0% incline. After 1 min, the grade was increased to 2.0%. At the start of the 3rd min and each minute thereafter, the grade was increased by 1%. The same speed was maintained throughout. The test was stopped when the subject self-reported worsening of concussive symptoms, when they reached HR max for age or at 20 min, and HR was recorded using pulse oximetry. Participants who reached either the HR max for age or completed 20 min on the treadmill were excluded from the study. For the remaining participants, the HR at which they reported worsening was defined as the threshold HR and used to guide the intensity of exercise for the intervention arm.

### Outcome Assessment

#### Feasibility and Acceptability

1. **Enrollment:** Recorded numbers of patients screened, eligible, and enrolled.
2. **Retention/attrition:** Tracked rates of participant withdrawal and loss to follow up. Recorded reasons for withdrawal, and tracked adverse events.
3. **Engagement:** Recorded visit completion rates, and rates of completion of surveys and other procedures.
4. **Satisfaction:** Subjects completed an end of study interview regarding satisfaction with study procedures and suggestions for improvement.
5. **Safety:** RA called subjects weekly to track exercise tolerance and any adverse events.

### Preliminary Efficacy Outcomes

**Trajectory of concussive symptoms, measured using the Health behavior inventory** (5, 36)**:** The HBI is a 20-item instrument assessing post-concussive symptoms on a four-point Likert scale with higher scores indicating greater severity. This scale has demonstrated validity and reliability among adolescents with mild traumatic brain injury (mTBI) (36, 37). The HBI was measured at nine time points: Baseline, weekly during the intervention (weeks 1–6), 3 months, and 6 months.

### Exploratory Outcomes

1. **Trajectory of Health-related quality of life, measured using the Pediatric Quality of Life Inventory (youth and parent report):** PedsQL© is a 23-item five point questionnaire that assesses physical, emotional, social, and school functioning, with established validity and reliability (29, 38). PedsQL was measured at four time points: Baseline, 6 weeks, 3 months, and 6 months.
2. **Change in physical activity, measured using accelerometry**: Physical activity was measured pre- and post-intervention (baseline and 6 weeks) in both experimental arms using a hip-mounted ActiGraph GT3X (ActiGraph LLC, Pensacola, FL). The GT3X accelerometers collect data at a frequency of 30 Hz, and data was processed into 1 s epochs (39–41). Participants wore the accelerometer for 1 week at baseline and in the last week of the intervention. We used the accelerometer data quality standards by Troiano et al. (42), including criteria for wear time and valid days (4–7 days, eight or more hours of accelerometer wear/day) (42). We used the accelerometer cut-points for moderate-to-vigorous physical activity (MVPA) developed by Evenson et al. (43), which has the highest classification accuracy (44). Total minutes above the threshold was divided by number of valid days to obtain minutes of MVPA per day pre- and post-intervention.
3. **Trajectory of Fear-avoidance, measured using the Fear of pain questionnaire** (45): The FOPQ-C and FOPQ-P are 24- and 23-item child and parent proxy versions of the Fear Of Pain Questionnaire, and have been shown to reliably and validly measure pain-related fear and pain-related avoidance of activities in youth (Cronbach's alpha 0.92) (23, 45). Fear-avoidance was measured at four time points: Baseline, 6 weeks, 3 months, and 6 months.

### Analysis

#### Descriptive

Participant characteristics, including sociodemographic characteristics, were summarized overall and compared by study arm using chi-square. Continuous measures were summarized via means and standard deviations, or median and interquartile range for asymmetrically distributed variables, and were compared by Student's *T*-tests and Wilcoxon rank sum, respectively.

#### Feasibility and Acceptability

Feasibility and acceptability measures were descriptively summarized. Satisfaction was measured qualitatively and themes were summarized.

#### Preliminary Efficacy Outcome

We collected pilot data regarding trajectory of concussive symptoms (HBI), hypothesizing that intervention youth would have a more rapid decline in concussive symptoms than control youth. Our previous work (13) has suggested that injury recovery follows an exponential curve with a rapid rate of decline initially after injury, and thus we modeled trajectory of concussive symptoms using an exponential decay framework which fit well with the data.

$$\begin{array}{l} HBI\left(t\right)=Nbl^{*}e^{-\lambda t} \end{array}$$

In this model, N_BL_ represents mean HBI at study baseline, estimated as a linear function of random intercept (HBI at the time of injury, N_0_) and covariates (duration of symptoms at entry into the study, age at concussion, and sex):

$$N_{BL}=N_{0}+\beta X.$$

We modeled intercept as a random effect, modified based on factors that might impact concussive symptoms at study baseline (sex, age, prior concussion, and duration of symptoms). Rate of exponential decay (non-linear slope) was modified based on covariates expected to impact rate of symptom resolution (prior concussion, duration of symptoms). Rate of change at time t weeks from study baseline, denoted by -λ_t_, was estimated as a linear function of study arm and covariates:

$$\lambda_{t}=\gamma_{0}+\gamma_{1}(Intervention)+\gamma X.$$

A statistically significant intervention effect (λ_1_) therefore denotes a difference in the rate of symptom resolution, accounting for duration of symptoms at entry into the study, age, sex, and prior concussion.

### Exploratory Outcomes (MVPA, Fear-Avoidance and Health-Related Quality of Life)

Given skewness in MVPA data, we examined this outcome categorically, assessing the proportion in each group who achieved a minimum of 15 min per day of MVPA at 6 weeks with logistic regression. Fear-avoidance (FOPQ) and health-related quality of life (PedsQL©), were entered into separate linear mixed models. Models were covariate-adjusted for duration of symptoms at entry into the study, age, sex, and prior concussion. Within participant correlation was modeled via an autoregressive covariance structure.

## Results

### Sample Description

Half of the participants were female, with average age 15.5 years (SD 1.6, Table 1). Approximately half the sample reported a prior concussion. Duration of concussion symptoms at the start of the study averaged about 2 months, but was quite skewed (median 48.5 days, IQR 33, 64). Concussions occurred from a wide range of sports (Table 1). The majority of youth (70%) reported headaches “often” at study entry. Approximately 40% had difficulty with concentrating and being tired. Only 17% reported history of chronic pain, but 73% had pain in the week before starting the study, all of whom had headaches. A smaller proportion reported neck pain (33%), back pain (23%), or pain in other locations (17%). Half the subjects reported family history of chronic headaches, 60% back or neck pain, and 47% other joint pain.

**Table 1:** Demographic characteristics of concussed youth participating in Sub-Threshold Exercise Program (STEP) study, Seattle WA 2016–17.

	**Control (*n* = 11) *Mean (SD) or N (%)***	**Intervention (*n* = 19) *Mean (SD) or N (%)***	**Total (*n* = 30) *Mean (SD) or N (%)***
Female	6 (54.6)	12 (63.2)	17 (56.7)
Age at baseline (years)	15.8 (1.1)	15.4 (1.8)	15.5 (1.6)
**Race** ^**a**^			
White	7 (63.7)	17 (89.5)	24 (82.8)
African-American/Black	2 (18.2)	–	2 (6.6)
Asian	–	2 (10.5)	2 (6.6)
Missing	2 (18.2)	–	2 (6.6)
**Ethnicity**			
Hispanic	–	1 (5.2)	1 (2.9)
Non-hispanic	10 (90.9)	17 (89.5)	31 (88.6)
Missing	1 (9.1)	1 (5.3)	2 (6.7)
**Household income**			
$0–60,000/year	3 (27.3)	3 (15.8)	6 (20.0)
History of prior concussion	7 (63.6)	8 (42.1)	15 (50.0)
Duration of symptoms at baseline (days) ^b^	75.9 (49.4) Range 22–202	48.8 (32.2) Range 21–175	58.8 (41.1) Range 21–202
Mechanism of injury			
Football	1 (09.1)	4 (21.1)	5 (16.7)
Soccer	2 (18.2)	3 (15.8)	5 (16.7)
Basketball	3 (27.3)	1 (5.2)	4 (13.3)
Wrestling	1 (9.1)	2 (10.5)	3 (10.0)
Swimming	–	2 (10.5)	2 (6.7)
Volleyball	–	2 (10.5)	2 (6.7)
Softball	1 (9.1)	1 (5.2)	2 (6.7)
Lacrosse	1 (9.1)	–	2 (6.7)
Tennis	1 (9.1)	–	1 (3.3)
Hockey	–	1 (5.2)	1 (3.3)
Ultimate frisbee	1 (9.1)	–	1 (3.3)
Gymnastics	–	1 (5.2)	1 (3.3)
Dance	–	1 (5.2)	1 (3.3)

a *Chi-square, p = 0.06*.

b *Wilcoxon rank sum, p = 0.02*.

#### Feasibility and Acceptability

Two participants discontinued the study after randomization but prior to starting the intervention as their symptoms had already resolved. Retention was excellent for participants who began the intervention, with 90% of youth and 83% of parents who began the intervention completing 6 month follow up. During interviews with parents and youth, 100% reported they would recommend the study to a friend. Other themes included: (1) enjoyment of the study (particularly incentives, weekly RA check-ins and use of wrist-based heart rate monitors) (2) appreciation of structure and organization, and (3) appreciation of minimal in-person study visits. Symptoms during exercise were reported by 2/11 subjects in the control group (18.2%) and 7/19 (36.8%) subjects in the intervention group, but were managed effectively by decreasing intensity of exercise, and no subjects chose to leave the study due to symptom exacerbations. Most symptom exacerbations occurred in the first few weeks of the study. Two individuals in the intervention group had symptoms exacerbate in later weeks, both of which were associated with participants engaging in a longer duration of exercise than was prescribed. No adverse events occurred during the study. We did not inquire as to whether participants were able to return to sporting activities.

### Quantitative Outcomes

**Figure 3:**
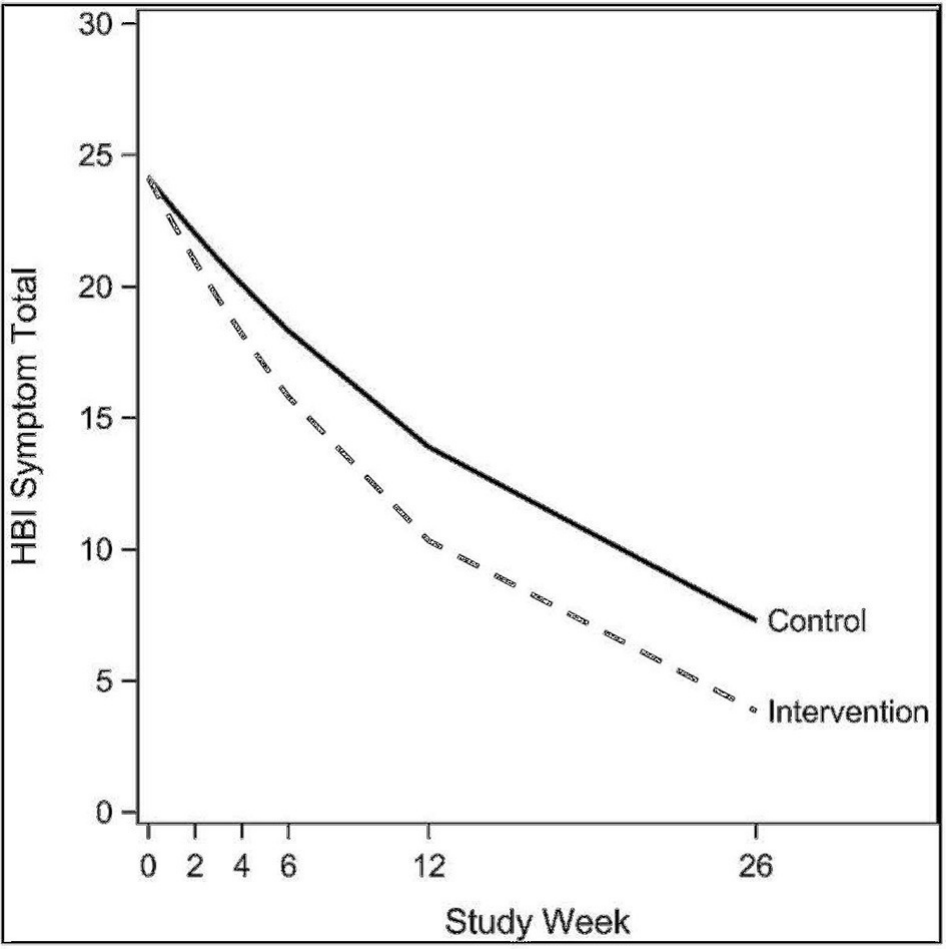
Exponential decay models examining the effect of the Sub-Threshold Exercise Program (STEP) on concussive symptoms in youth, Seattle WA 2016–17.

**Figure 4:**
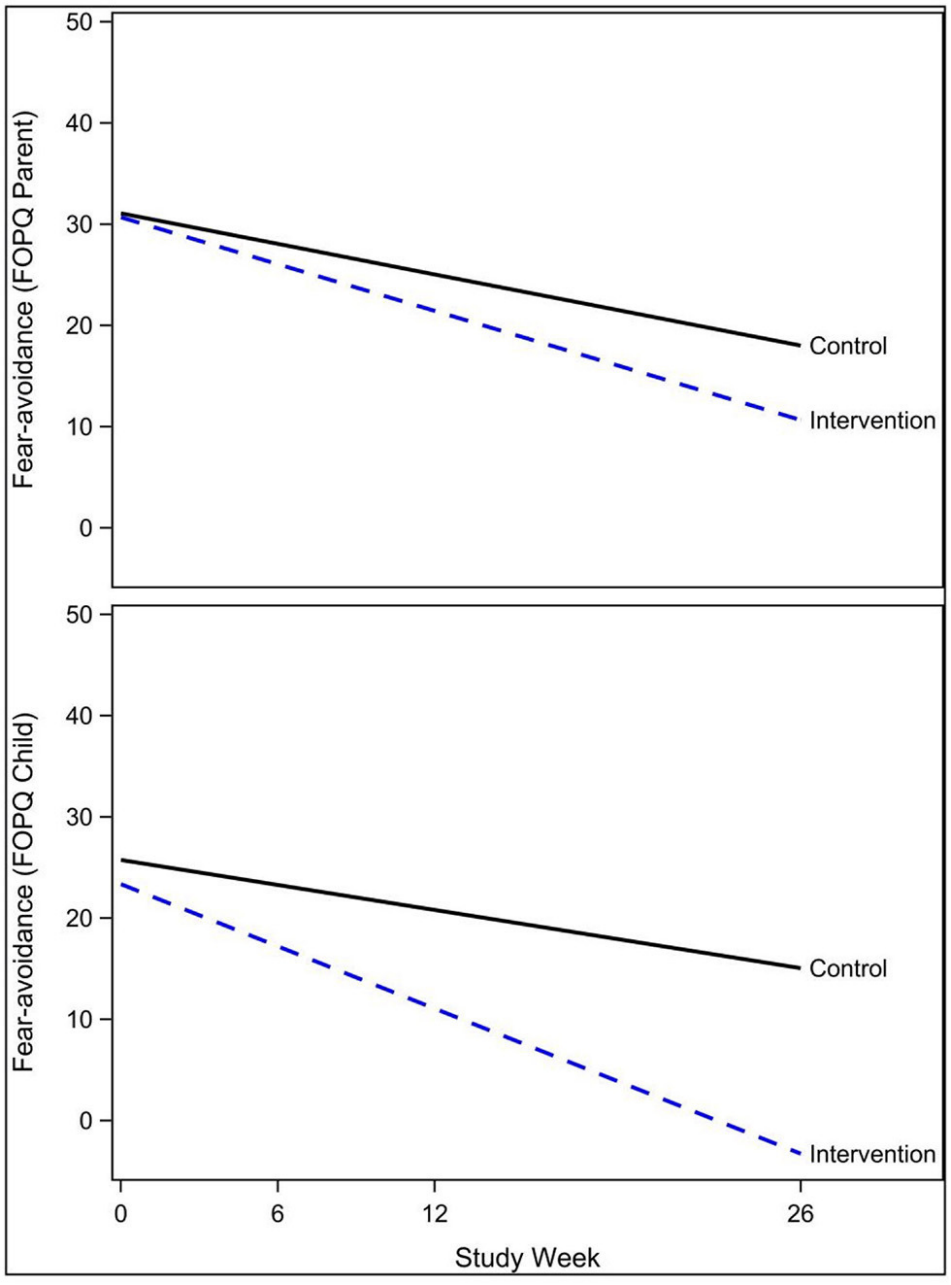
Effect of the Sub-Threshold Exercise Program (STEP) on fear-avoidance in youth, Seattle WA 2016–17.

The exponential decay model revealed a significant effect of the intervention on rate of decline of concussive symptoms (HBI, *p* = 0.02, Table 2 and Figure 3), after controlling for age, sex, and prior concussion. Rate of concussion symptom improvement was slower among youth with chronic symptoms (9–22 weeks, and >22 weeks) compared to those with acute symptoms (<9 weeks) in both intervention and control groups. However, the intervention effect (i.e., the difference in rates between intervention and control youth) was also most pronounced in youth with chronic symptoms. All subjects completed the pre-intervention accelerometry as it was a requirement for starting the study. Most subjects (26/30, 87%) completed the post-intervention accelerometry. The proportion of youth achieving a minimum of 15 min/day of MVPA was not significantly different by study arm. Parent-reported health-related quality of life (PedsQL©) was significantly improved in intervention compared to control youth (β = 0.56, *p* = 0.05; Table 3). Child-reported PedsQL© improved overall (β = 0.75, *p* = 0.0045; Table 3), but was not significantly affected by the intervention (β = 0.19, *p* = 0.47; Table 3). Parental fear-avoidance (FOPQ-P) significantly declined overall (*p* = 0.0096), but was not different by treatment group (β = −0.0031, *p* = 0.99, Table 3; Figure 4). Child fear-avoidance (FOPQ-C) was not significantly different by treatment group (β = −0.29, *p* = 0.23, Table 3; Figure 4).

**Table 2:** Exponential decay model examining the effect of the Sub-Threshold Exercise Program (STEP) on concussive symptoms over time (Health Behavior Inventory, or HBI), adjusted for age, sex, duration of time since injury and history of concussion, Seattle WA 2016–17.

		**Estimate**	**95% Confidence limits**		***p*-value**
Intercept	mu	24.48	−27.60	76.56	0.36
Age (continuous)	c1	0.69	−2.63	4.01	0.69
Female	c2	−4.68	−15.55	6.19	0.40
Duration of symptoms					
<9 weeks (referent)	–				
9–22 weeks (int)	b2	−8.24	−12.85	−3.62	**0.0005**
>22 weeks (int)	b3	−3.97	−11.65	3.71	0.31
Rate	g0	0.12	0.05	0.19	**0.0009**
Duration of symptoms					
<9 weeks (referent)	–				
9–22 weeks (rate)	g2	−0.07	−0.14	0.00	**0.048**
>22 weeks (rate)	g3	−0.03	−0.06	−0.01	**0.01**
Prior concussion (y/n)	c3	−0.01	−0.03	0.01	0.25
Intervention	g1	0.02	0.00	0.04	**0.02**

*Bold values indicate the highlighted results that are significant with p values <0.05*.

**Table 3:** Linear mixed model examining the impact of the Sub-Threshold Exercise Program (STEP) intervention on concussed youth on outcomes of fear avoidance of pain and health-related quality of life, adjusted for age, sex, duration of symptoms, and prior concussion, Seattle WA 2016–17.

	FOPQ^a^ (Child)			FOPQ^a^ (Parent)			PEDsQL© ^b^ (Child)			PEDsQL© ^b^ (Parent)		
	B	(SE)	p	B	(SE)	p	B	(SE)	p	B	(SE)	p
Mean												
Intercept	−24.54	(28.54)	0.40	20.80	(28.80)	0.48	71.90	(22.91)	0.0044	57.97	(24.22)	0.02
Age (continuous)	3.24	(1.88)	0.10	1.03	(1.90)	0.59	−0.10	(1.51)	0.95	−0.06	(1.60)	0.97
Female	13.64	(5.45)	**0.02**	9.58	(5.42)	0.59	−6.49	(4.35)	0.15	−1.21	(4.53)	0.79
Prior concussion	−8.79	(6.02)	0.16	−9.02	(6.03)	0.15	−1.18	(4.84)	0.81	7.73	(5.12)	0.14)
Duration of symptoms												
*<9 weeks (referent)*	–	–	–	–	–	–	–	–	–	–	–	–
*9–22 weeks*	−7.26	(4.73)	0.13	−5.83	(4.49)	0.20	15.46	(3.04)	**<0.0001**	11.42	(3.49)	**0.0016**
*>22 weeks*	4.31	(3.62)	0.24	6.35	(3.33)	0.06	−11.46	(3.95)	**0.0048**	−0.82	(4.49)	0.86
Slope												
Week	−0.40	(0.26)	0.13	−0.63	(0.24)	**0.0096**	0.75	(0.26)	**0.0045**	0.22	(0.29)	0.44
Week x Intervention	−0.29	(0.25)	0.23	−0.0031	(0.23)	0.99	0.19	(0.25)	0.47	0.56	(0.29)	**0.05**

a *Fear of Pain Questionnaire, a measure of fear-avoidance of pain*.

b *Pediatric Quality of Life Inventory, a measure of health-related quality of life.*.

*Bold values indicate the highlighted results that are significant with p values <0.05*.

## Discussion

While previous studies utilized sub-threshold exercise to treat youth with persistent concussion, this is the first study to measure MVPA using accelerometry, and the first to examine an intervention requiring minimal in-person visits. This is also the first study to explore the impact of exercise on fear-avoidance of pain in youth with concussion. We found the Sub-Threshold Exercise Program (STEP) was feasible and acceptable for youth with concussive symptoms. Youth reported enjoying the structure and support provided, and preferred the minimal visits. Preliminary data suggested potential benefit of this approach, as youth who received STEP had more rapid improvement in concussion symptoms compared to youth in a stretching control, and improvement was maintained at 6 months. Health-related quality of life improved significantly for all subjects and fear-avoidance of pain declined significantly for all subjects, but neither was significantly different by intervention group.

Our findings are in line with prior studies of exercise interventions for concussed youth, all of which report benefit (8–15). Much of our methodology was similar to prior studies: (a) utilizing the Buffalo Concussion Treadmill Test to determine HR threshold, (b) setting exercise goals at 80% of the HR threshold (8, 9), and (c) asking participants to exercise for 6 weeks (8–15). However, previous studies of sub-threshold exercise required weekly in-person visits, which present greater barriers for access and are difficult to scale. Instead, we utilized RAs as health coaches, phoning youth weekly to advance exercise goals, and required only two in-person visits, which we felt was a more scalable approach. We also provided youth with wrist-mounted HR monitors, to allow them to self-monitor HR goals. In addition, we objectively measured MVPA using hip-mounted accelerometers pre- and post-intervention, which allowed us to tailor the intervention to activity level, ensuring that the intervention was an increase from baseline MVPA. Utilizing accelerometry also allowed us to quantify changes in MVPA. Despite the impact of the intervention on concussive symptoms, it did not significantly change MVPA compared to controls in this pilot study.

Finding no significant change in MVPA and yet a positive effect of the intervention on rate of decline of concussive symptoms poses a new question—how can an aerobic exercise intervention be efficacious if it does not increase MVPA? We suggest caution in examining these MVPA data, given the relatively small sample size. In addition, accelerometry is an imperfect measure of MVPA, and certain types of physical activity such as swimming and cycling are not well-captured. The intervention and control groups also had differences in amount of MVPA at baseline, which makes it challenging to measure improvement. The lack of differences in MVPA does suggest we should consider other factors. In planning this study, we felt it would be beneficial to examine concepts from the chronic pain literature, given the prevalence of headache as a chronic symptom of concussion. One prevalent theory regarding the development of chronic pain is fear-avoidance of pain (18–22).

Prior studies (25–27) have measured high levels of fear-avoidance of pain and fear of physical activity (“kinesiophobia”) in patients with persistent concussive symptoms. However, this is the first study to report decreases in fear-avoidance of pain in patients undergoing two different exercise treatments for concussion, and this finding deserves further study. Clearly further work needs to be done to examine the relationship between fear-avoidance and concussion symptom resolution longitudinally, as there are possible bidirectional relationships. In addition, “fear-avoidance” contains multiple concepts—pain-catastrophizing, fear of pain, and avoidance of activities that might result in pain. Future studies should disentangle these concepts, and better understand potential predictive relationships between children's psychological and behavioral response to concussion and their subsequent symptom experience using mediation analyses. It is of note that the intervention was most efficacious for youth with persistent symptoms (>9 weeks), fitting with a chronic pain model (18–22). Additional research is needed to explore whether mediators of exercise treatment effects vary with chronicity of symptoms.

It is notable that more than half of the youth in our study had parents who reported chronic pain. Parents play an important role in contextualizing pain and other negative symptoms for their children (23, 46–48). If a parent expresses fear about a child's pain, their child is more likely to be concerned about potential danger. Intervening with parents to decrease pain catastrophizing, pain fears and avoidance of potentially painful activities has proven beneficial for youth with chronic pain (49–51). While the STEP intervention included parents at the baseline visit, parents did not receive tailored education and were not significantly involved in weekly calls. Future studies might consider greater engagement with parents to help them support their child's exercise and lessen fear of concussive symptoms.

This study was limited by a lack of diversity, which affects the ability to generalize to other populations. We also had a small sample size and thus lacked the power to conduct a definitive assessment of efficacy or true mediation analysis regarding fear-avoidance of pain and MVPA, and future studies are needed to explore both of these areas. Hip-mounted accelerometry is also an imperfect measure of MVPA, and does not accurately capture a full range of activities such as swimming and cycling. Accelerometers are also relatively burdensome to wear, and must be used for brief periods of time, resulting in activity bias. We attempted to defray such issues through standardized methods including recommendations regarding wear time, but the data remain imperfect. Finally, we had no direct measure of adherence such as a daily log, which would allow us to better estimate compliance with the exercise intervention.

In conclusion, this study found that a Sub-threshold Exercise Program (STEP) for youth with persistent concussive symptoms was feasible, and may increase the rate of improvement in concussive symptoms. The STEP intervention was acceptable to subjects even with a small number of in-person visits, and treatment benefits were maintained at 6 month follow-up. Future studies are needed to examine this type of intervention in a larger sample powered to assess efficacy and mediation of treatment effects.

## Data Availability

The datasets generated for this study are available on request to the corresponding author.

## Author Contributions

SC conceptualized and designed the study, oversaw data analysis, drafted the initial manuscript and incorporated all revisions and comments. KW conducted data analysis, prepared the figures and reviewed and revised the manuscript. JM and FR assisted with conceptualization and design of the study, provided input regarding analysis, and critically reviewed the manuscript for important intellectual content. MB assisted with conceptualization and design of the study, and reviewed and revised the final manuscript. ES assisted with conceptualization and design of the study, assisted with data collection and reviewed and revised the final manuscript. AH and LF assisted with data collection and analysis, and reviewed and revised the final manuscript. TP provided input regarding analysis, and critically reviewed the manuscript for important intellectual content. All authors approved the final manuscript as submitted and agree to be accountable for all aspects of the work.

### Conflict of Interest Statement

The authors declare that the research was conducted in the absence of any commercial or financial relationships that could be construed as a potential conflict of interest.
