# Corrigendum: Pilot Randomized Controlled Trial of an Exercise Program Requiring Minimal In-person Visits for Youth With Persistent Sport-Related Concussion

**DOI:** 10.3389/fneur.2020.00006

**Published:** 2020-02-21

**Authors:** Sara P. D. Chrisman, Kathryn B. Whitlock, Jason A. Mendoza, Monique S. Burton, Ellie Somers, Albert Hsu, Lauren Fay, Tonya M. Palermo, Frederick P. Rivara

**Affiliations:** ^1^Center for Child Health, Behavior and Development, Seattle Children's Research Institute, Seattle, WA, United States; ^2^Department of Pediatrics, University of Washington, Seattle, WA, United States; ^3^Harborview Injury Prevention and Research Center, Seattle, WA, United States; ^4^Department of Orthopedics and Sports Medicine, Seattle Children's Hospital, Seattle, WA, United States; ^5^Department of Sports Physical Therapy, Seattle Children's Hospital, Seattle, WA, United States; ^6^Department of Anesthesiology and Pain Medicine, University of Washington, Seattle, WA, United States

**Keywords:** brain concussion, child, fear-avoidance, pain, exercise, traumatic brain injury, treatment, sport

In the original article, there was an error. In the **Methods** section, subsection **Preliminary Efficacy Outcome** the first formula was incorrectly written as:

$$\begin{array}{l} HBI_{\left(\text{t}\right)}={N_{\text{BL}}}^{\text{e}-\lambda t} \end{array}$$

This equation should read:

$$\begin{array}{l} HBI\left(t\right)=Nbl^{*}e^{-\lambda t} \end{array}$$

The authors apologize for this error and state that this does not change the scientific conclusions of the article in any way. The original article has been updated.

