# Supplementary material for: Pilot Randomized Controlled Trial of an Exercise Program Requiring Minimal In-person Visits for Youth With Persistent Sport-Related Concussion
Source: Front Neurol. 2019 Jun 17;10:623. doi: 10.3389/fneur.2019.00623 (PMC6611408; doi:10.3389/fneur.2019.00623)
Supplement: Supplementary file 1 [file Data_Sheet_1.PDF]

Seattle Children's

Prepared by: Elissa Somers on Apr 01, 2015
Access Code: LYZEMCVD
URL: http://sc.medbridgego.com/

Supine Cervical Retraction with Towel

reps: 10 sets: 3 hold: 5 Weekly: 5x Daily: 1x

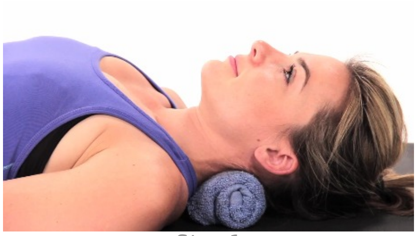
Step 1

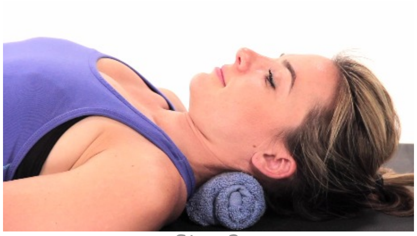
Step 2

Setup

- Begin lying on your back with a rolled towel positioned under your head.

Movement

- Gently tuck your chin as if you are making a double chin. Hold, then relax.

Tip

- Make sure not to lift your head from the rolled towel. You might feel a stretch along the back of your neck.

Seated Cervical Retraction

reps: 10 sets: 3 hold: 5 Weekly: 5x Daily: 1x

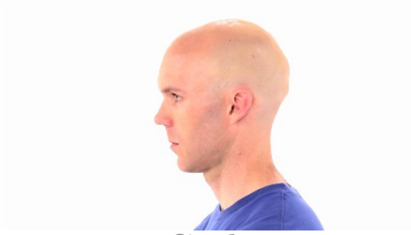
Step 1

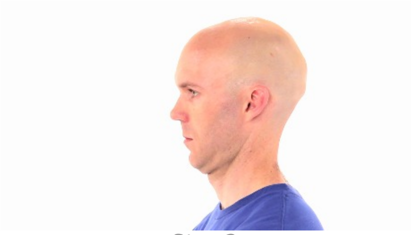
Step 2

Setup

- Begin sitting in an upright position with your feet flat on the floor.

Movement

- Gently draw your chin in, while keeping your eyes fixed on something in front of you.

Tip

- Make sure that you do not look down as you do this exercise, or bend your neck forward.

Prone Scapular Retraction

reps: 10 sets: 3 hold: 5 Weekly: 5x Daily: 1x

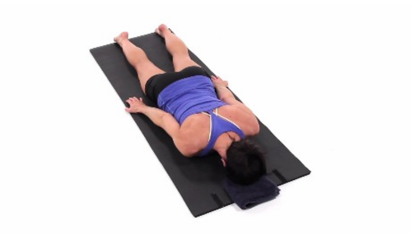
Step 1

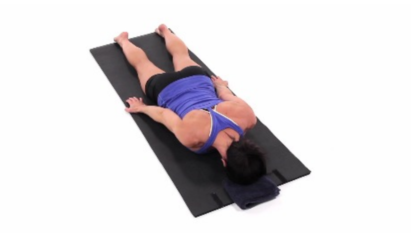
Step 2

Setup

- Begin lying face down with your arms resting to either side of your body.

Movement

- Gently squeeze your shoulder blades together, then relax them and repeat.

Tip

- Make sure to keep your back relaxed and do not shrug your shoulders

Prone Scapular Retraction Arms at Side

reps: 10 sets: 3 hold: 5 Weekly: 5x Daily: 1x

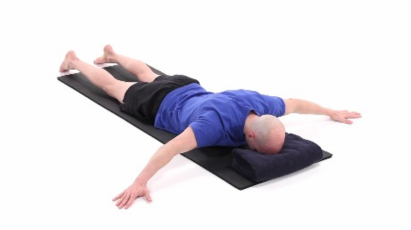
Step 1

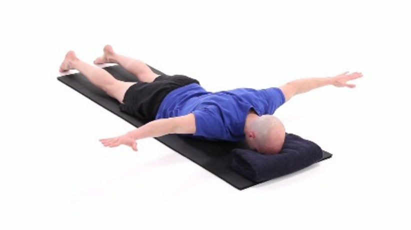
Step 2

Setup

- Begin lying face down with arms resting on the ground straight to your sides.

Movement

- Lift your arms toward the ceiling keeping your elbows straight.

Tip

- Think of squeezing your shoulder blades together as you lift your arms.

Supine Transversus Abdominis Bracing - Hands on Stomach

reps: 10 sets: 3 hold: 5 Weekly: 5x Daily: 1x

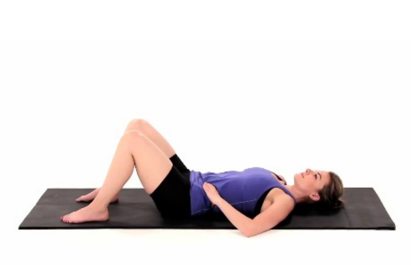
Step 1

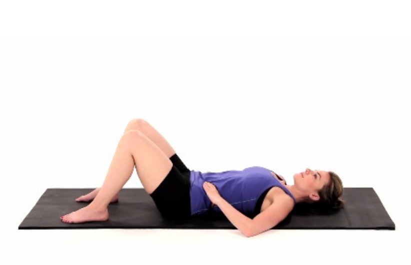
Step 2

Setup

- Begin lying on your back with your knees bent, feet resting on the floor, and your fingers resting on your stomach just above your hip bones.

Movement

- Tighten your abdominals, pulling your navel in toward your spine and up. You should feel your muscles contract under your fingers. Hold this position, then relax and repeat.

Tip

- Make sure to keep your back flat against the floor and do not hold your breath as you tighten your muscles.

Supine Single Bent Knee Fallout
reps: 10 sets: 3 hold: 5 Weekly: 5x Daily: 1x

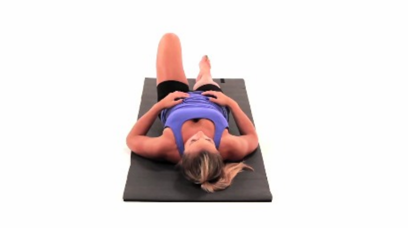

Step 1

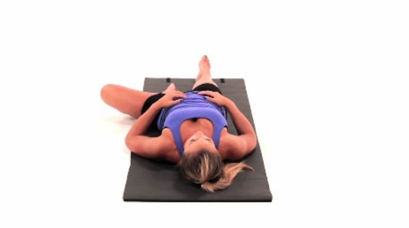

Step 2

Setup

- Begin lying on your back with one leg straight and your other knee bent.
  Movement

Movement

- Tighten your abdominals and slowly lower your bent knee toward the ground, then bring it back to the starting position, and repeat.

Tip

- Make sure to keep your abdominals tight, low back flat against the ground, and do not let your trunk rotate.
